# Evaluation of Articular Cartilage Regeneration Properties of Decellularized Cartilage Powder/Modified Hyaluronic Acid Hydrogel Scaffolds

**DOI:** 10.1021/acsomega.4c01927

**Published:** 2024-07-27

**Authors:** Paula Carmela O. Ching, Fang-Hsu Chen, I-Hsuan Lin, Duong-Thuy Tran, Lemmuel L. Tayo, Ming-Long Yeh

**Affiliations:** † Department of Biomedical Engineering, National Cheng Kung University, Tainan 701, Taiwan; ‡ School of Chemical, Biological, and Materials Engineering and Sciences, Mapua University, Manila 1002, Philippines; § Department of Biology, School of Medicine and Health Sciences, Mapua University, Makati 1205, Philippines; ∥ Medical Device Innovation Center, National Cheng Kung University, Tainan 701, Taiwan

## Abstract

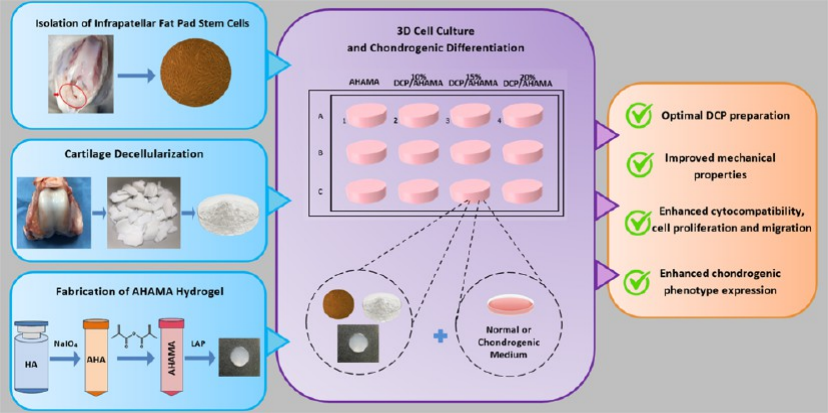

The articular cartilage has poor intrinsic healing potential, hence, imposing a great challenge for articular cartilage regeneration in osteoarthritis. Tissue regeneration by scaffolds and bioactive materials has provided a healing potential for degenerated cartilage. In this study, decellularized cartilage powder (DCP) and hyaluronic acid hydrogel modified by aldehyde groups and methacrylate (AHAMA) were fabricated and evaluated in vitro for efficacy in articular cartilage regeneration. In vitro tests such as cell proliferation, cell viability, and cell migration showed that DCP/AHAMA has negligible cytotoxic effects. Furthermore, it could provide an enhanced microenvironment for infrapatellar fat pad stem cells (IFPSCs). Mechanical property tests of DCP/AHAMA showed suitable adhesive and compressive strength. IFPSCs under three-dimensional (3D) culture in DCP/AMAHA were used to assess their ability to proliferate and differentiate into chondrocytes using normal and chondroinductive media. Results exhibited increased gene expression of COL2 and ACN and decreased COL1 expression. DCP/AHAMA provides a microenvironment that recapitulates the biomechanical properties of the native cartilage, promotes chondrogenic differentiation, blocks hypertrophy, and demonstrates applicability for cartilage tissue engineering and the potential for clinical biomedical applications.

## 1 Introduction

The articular cartilage has poor self-healing potential^1^ as it is an avascular tissue, has low cellular metabolic activity, and lacks access to stem cells.^2^ These characteristics pose a great challenge for articular cartilage regeneration. Several treatment methods have been widely investigated but the best available treatment for chondral and osteochondral defects remains debatable.^3^ Common treatment methods for articular cartilage defects include surgical treatment options such as microfracture, osteochondral autograft transplant (OAT), autologous matrix induced-chondrogenesis (AMIC), and autologous chondrocyte implantation (ACI).^4,3^ With the limitations to current treatment methods, a promising viable alternative is the field of articular cartilage tissue engineering, which aims to repair, restore, and improve damaged articular cartilage.^5^ A recent study presented the potential use of tissue engineering to provide alternative treatment by utilizing cell-based therapy with ECM substitutes and bioactive molecules to prepare functional tissue replacement of hyaline cartilage.^6^

The production of engineered tissue in vitro requires the use of cells, usually taken from the patient, in conjunction with scaffolds to produce a matrix resembling native tissue. However, the collection of cells poses limitations, such as invasiveness and diseased cell state. Therefore, attention has become focused on the use of stem cells, including embryonic stem (ES) cells, bone marrow mesenchymal stem cells (BM-MSCs), and umbilical cord-derived mesenchymal stem cells (UC-MSCs).^7^ For cartilage repair, the extensively explored sources of chondrogenic cells are chondrocytes and mesenchymal stem cells (MSC).^8^ One emerging source of MSCs is the infrapatellar fat pad (IFP) tissues^9,10^ which can be harvested from the articular knee joint to isolate the IFPSC. This source is known to have high chondrogenic potential,^11^ even higher than other MSCs sourced from other tissues due to its proximity to the knee joint^12^ and is, therefore, used in cartilage tissue engineering. Moreover, IFP can be easily and safely harvested during routine surgical procedures, such as arthroplasty and arthroscopy.

Using biomaterial scaffolds in tissue engineering can provide an environment that mimics the native ECM, structural support, and a venue for cellular adhesion and differentiation.^13^ The ideal scaffold for cartilage tissue engineering must possess the following desired properties: biodegradable, nontoxic, favorable resorption kinetics, should not hinder the ability to fix to the defect site, supports cell attachment and directs cell expression, allows cell migration, and facilitates the transport of nutrients.^14^ A scaffold must mimic the ECM by exhibiting the biological, chemical, and mechanical cues that influence cell phenotype and tissue formation.^15^ The use of decellularized extracellular matrix (dECM) proved to be beneficial as it retains the natural ECM environment^16^ and low immune response because of the removal of cellular DNA.^17^ Current decellularization processes, though resulting in reduced immune reaction, also reduce sulfated glycosaminoglycans (sGAG),^18−20^ collagen content,^21^ as well as biomechanical properties.^20^ Though there is no standard protocol, optimal decellularization methods should effectively remove cellular components while preserving collagen, glycosaminoglycans (GAGs), and growth factors, and maintain the ECM ultrastructure and micromechanical properties.^22^

The emerging use of hydrogels has made progress in cartilage tissue engineering,^23,24^ and other clinical applications.^25^ Hyaluronic acid (HA) is a natural glycosaminoglycan found in many tissues, including cartilage.^26^ HA is widely used in cartilage tissue engineering since it is known to have good biocompatibility, promote the proliferation of chondrocytes^27^ and MSCs,^28^ and promote cartilage regeneration.^29^ However, HA-based hydrogels have high degradation rates and unfavorable mechanical properties, limiting their application in cartilage tissue engineering.^30^ Previous researches have utilized HA in conjunction with other natural biomaterials such as collagen for cartilage regeneration^31^ or synthetic polymers^32^ in order to improve their biological and mechanical properties. Furthermore, HA can be chemically modified to tailor its properties for preclinical and clinical applications.^33^ Several chemical modifications can be performed on HA since it has three targeted sites for modification: the carboxyl group, hydroxyl group, and −NHCOCH_3_. These modifications improve mechanical properties, degradation, viscosity, solubility, and biological properties,^34^ thereby, enhancing their suitability for cartilage tissue engineering applications.^30^ Methacrylation of HA (HAMA) has been shown to enhance resistance to enzymatic degradation^35^ and improve the mechanical and physical properties of HA.^36^ Another modification involves the addition of aldehyde groups, which improves its adhesive performance.^37^ In a recent study, a modified HA methacrylate hydrogel with aldehyde groups (AHAMA) was successfully developed. By incorporating methacrylate, the hydrogel’s stability was improved, and the addition of aldehydes provided additional anchoring mechanisms, enhancing the gel’s adhesion to the native cartilage.^38^

DCP and AHAMA, separately, have been proven to be effective in cartilage regeneration in vivo. However, in order to overcome their individual limitations such as structure and applicability, mechanical properties, and adhesion to native tissue, this research utilized a combination of these two. In this study, decellularized porcine articular cartilage powder (DCP) and AHAMA were used as scaffold materials to provide the structure, composition, architecture, and function of the ECM. Through the incorporation of DCP and AHAMA, this study investigated the ability to provide an adequate environment for the proliferation and migration of cells, mechanical properties, and suitability of the scaffolds to recapitulate the complex biomechanical properties of native cartilage in an in vitro setting. Using cell encapsulation, this study aims to find a suitable concentration of DCP/AHAMA combination that can express the chondrogenic phenotype without the aid of additional signaling factors.

## 2 Materials and Methods

### 2.1 Materials

Infrapatellar fat pad (IFP) tissues were harvested from adult New Zealand white rabbits undergoing total knee arthroplasty at the Laboratory Animal Center, National Cheng Kung University (NCKU LAC). Fresh porcine knees were obtained from a local abattoir in Tainan, Taiwan. Sodium hyaluronate (MW 50,000–200,000, 0911AP, AK Scientific, Inc., California), sodium periodate (7790-28-5, Thermo Fisher Scientific Inc.), ethylene glycol (ED0199, Bio Basic, Canada), methacrylic anhydride (760-93-0, Sigma-Aldrich), lithium phenyl-2,4,6-trimethybenzoylphosphinate (LAP, 85073-19-4, Sigma-Aldrich), and other commercially available reagents were used as received.

**Figure 1:**
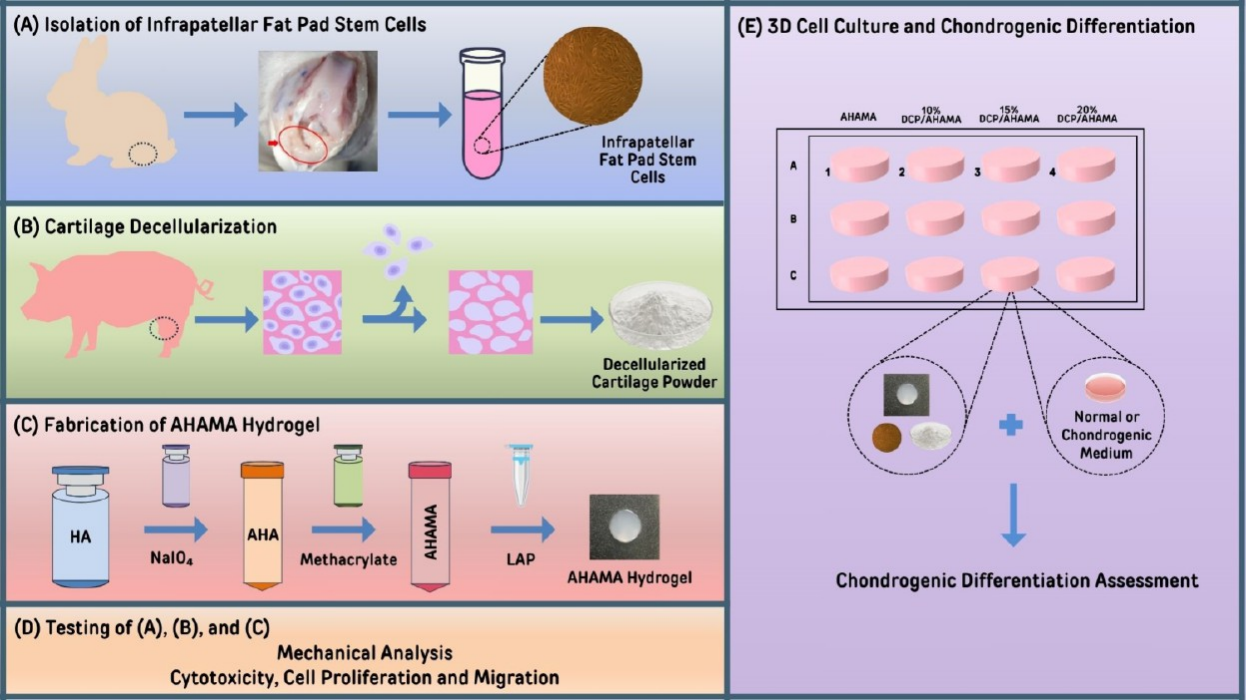
Schematic diagram of the overall system. (A) Isolation of infrapatellar fat pad stem cells, (B) preparation of decellularized cartilage, (C) fabrication of AHAMA hydrogel, and (D) testing of DCP and AHAMA alone or in combination for mechanical properties and cytotoxicity, cell proliferation, and migration. (E) 3D cell culture of IFPSCs in AHAMA or varying concentrations of DCP/AHAMA.

The experiment utilized three main components: IFPSCs, DCP, and AHAMA, which were prepared separately as shown in Figure 1A–1C. Then, AHAMA or DCP/AHAMA in combination were tested for mechanical properties, and DCP or DCP/AHAMA in combination were tested for cytotoxicity, cell proliferation, and migration (Figure 1D). Lastly, three-dimensional (3D) cell culture where AHAMA or various concentrations of DCP/AHAMA were assessed for their ability to influence the chondrogenic phenotype expression of IFPSCs (Figure 1E).

### 2.2 Isolation of Infrapatellar Fat Pad Stem Cells

The harvested IFP tissues were placed in 3 mL of Dulbecco’s modified Eagle’s medium-low glucose (DMEM-LG; Gibco) supplemented with 10% fetal bovine serum (FBS) and 1% antibiotic/antimycotic (AA) and transported back to the lab for immediate processing. In brief, the tissues were washed with 1× phosphate buffer solution (PBS) to remove possible contaminants and excess oil. The tissues were then chopped into small pieces of about 0.5 mm^3^ and added into 0.2% collagenase (20 mg collagenase/10 mL DMEM) in 15 mL conical tubes to hydrolyze native collagen, detaching the primary cell from the tissue. The tubes were incubated overnight at 37 °C and 5% CO_2_ on an orbital shaker. The tissues should be digested and homogenized the following day, then centrifuged at 400× for 10 min. The supernatant, containing adipocytes and oily fat, was discarded. The retained pellet was mixed with 10 mL of PBS, passed through a sterile filter (Cell Strainer), and centrifuged at the same parameters. The pellets were resuspended in 1 mL of DMEM, homogenized, and seeded into a 100 mm × 20 mm Petri dish containing 7 mL of DMEM (10% FBS, 1% AA). The dish was incubated for 24 h at 37 °C and 5% CO_2_, then washed with 1× PBS (1% AA) three times to remove oil. The medium was changed daily or every 2 days. When the cells reached ∼80–100% confluence, they were detached with 1× trypsin (Gibco, Canada) and recultured as the first passage with complete medium through the fifth passage. Only cells from passages 5–6 were used in succeeding stem cell experiments.

### 2.3 Cartilage Decellularization

Cartilage was harvested from adult pig knees and carefully removed using a scalpel. The cartilage was cut into smaller fragments, washed with PBS, and freeze-dried for 24 h. After the fragments were dry, a tissue homogenizer was used to grind the samples and the samples were sifted using a pore filter to obtain powders of about 250 μm diameter.

To decellularize about 5 g of cartilage powders, they were placed in a 50 mL tube to soak in various agents. First, 0.25% trypsin–ethylenediaminetetraacetic acid (EDTA) solution was added and placed in an orbital shaker for 24 h, changing the solution every 8 h. The suspension was centrifuged at 3000 rpm for 10 min, and the supernatant was discarded. The samples were washed with PBS for 30 min, then soaked in 10 mM Tris–HCl containing 50 u/mL DNase and 1 u/mL RNase for the next 4 h, and washed with the enzyme-free 10 mM Tris–HCl for another 20 h. The samples were centrifuged again, and the supernatant was discarded. 1% Triton X-100 solution was added and soaked for another 24 h. The samples were rinsed with PBS for 24 h using a shaker, changing the solution every 8 h. After centrifugation to discard the supernatant, the cartilage suspension was freeze-dried for 2 days to obtain decellularized cartilage powder. After decellularization, the native and decellularized cartilage tissues were stained with hematoxylin and eosin (H&E) and Masson’s trichrome stain.

To quantify the sulfated glycosaminoglycans (sGAG) that were retained after decellularization, the dimethyl methylene blue (DMMB) dye assay was performed. The O.D. values were measured at a wavelength of 562 nm under an enzyme-linked immunosorbent assay (ELISA) reader. The standard curve was plotted by preparing various concentrations of chondroitin-4-sulfate in DI water (0–500 μg/mL). To quantify the DNA removal in the samples, DNA was extracted before and after decellularization using Invitrogen TRIzol reagent, and the absorbance was measured using a NanoDrop spectrophotometer (Thermo Fisher Scientific Inc.).

### 2.4 Fabrication of AHAMA Hydrogel

AHAMA was synthesized according to the procedures performed by Tan et al. and Chen et al.^39,38^ 1 g of HA was dissolved completely in 100 mL of water, then 5 mL of 0.5 M sodium periodate was added slowly, and the reaction was kept for 2 h in the dark. To inactivate the unreacted periodate, 1 mL of ethylene glycol was added and allowed to react for 1 h. After dialysis for 3 days, AHA was obtained by freeze-drying. After the dried AHA was obtained, 1 g of AHA was completely dissolved into 100 mL of water. To modify AHA with double bonds, 1 mL of methacrylate was added, and the reaction was carried out for 12 h. pH was maintained at 8–8.5, and the whole process was carried out on ice. After the reaction, the solution was dialyzed for 2 days and freeze-dried. AHAMA was dissolved in PBS solution (3% w/v) with 0.3% (w/v) of the photoinitiator lithium phenyl-2,4,6-trimethylbenzoylphosphinate (LAP). Then, the hydrogel was formed by exposure to 405 nm ultraviolet (UV) light. The overall reaction for fabrication of synthetic AHAMA is shown in Figure 4. Characterization of AHAMA was performed with ^1^H NMR (Bruker-500).

#### 2.4.1 Swelling Ratio

Using a 7 mm × 4 mm (diameter × depth) mold, hydrogels of 180 μL volume were prepared by exposure to 405 nm UV light for 1, 3, and 5 min and dried overnight using a vacuum dryer. The dry weight was noted as *W*_d_. The samples were then soaked in PBS and incubated for various periods (10, 20, 30, 1, 5, 12, and 24 h) and then filtered and weighed (*W*_s_). The swelling ratio was calculated as follows
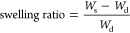
1

#### 2.4.2 Degradation Test

To measure the degradation rate, 180 μL of AHAMA hydrogel was prepared by 1, 3, and 5 min UV exposure and lyophilized overnight. The initial weight was measured and recorded as *W*_i_. Then, the samples were incubated at 37 °C in 2 mL of PBS or 2 mL of collagenase in PBS (0.5 mg/mL) to measure the enzymatic degradation for 1, 3, 7, 14, 21, and 28 days. At predetermined time points, the solutions were removed and the samples were lyophilized overnight to obtain the degraded weight (*W*_d_). The pH values were tracked every 7 days before being replaced with fresh solutions. The mass losses (%) of hydrogels were calculated using the following equation
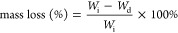
2

#### 2.4.3 Protein Adsorption

In a mold, 180 μL of AHAMA was prepared to fabricate the scaffolds and incubated at 37 °C in the medium containing 10% FBS for 2 h. The medium was removed, and the samples were rinsed with PBS three times, followed by soaking in 240 μL of 1% sodium dodecyl sulfate (SDS) solution for 2 h at room temperature. The SDS solution was collected into the vials, and fresh 240 μL of 1% SDS solution was added to the samples for another 2 h. All SDS solutions were merged and pipetted in a vial, and 150 μL from each group was drawn for reaction with the Micro BCA protein assay kit. The O.D. values were measured under an ELISA reader at 562 nm wavelength, and the standard curve was created by preparing different concentrations of commercial bovine serum albumin from 0 to 200 μg/mL.

### 2.5 Mechanical Analysis

#### 2.5.1 Morphological Evaluation

The scaffolds were observed in both gross and microscopic morphologies. Digital images were recorded to evaluate the gelation in a macroscopic view, and the microscale morphology was characterized using scanning electron microscopy (SEM; JEOL, JSM-6700F, Japan). The scaffolds were hemisected and then coated with gold for 300 s to investigate the porous structure inside.

The particle size, morphologies, and pore size of the DCP were also examined using SEM. The powder particles were fixed on double-sided carbon tape and then sputter-coated with gold for 300 s. Finally, it was observed under an SEM instrument operated at an acceleration voltage of 10.0 kV.

#### 2.5.2 Adhesion Test

**Figure 2:**
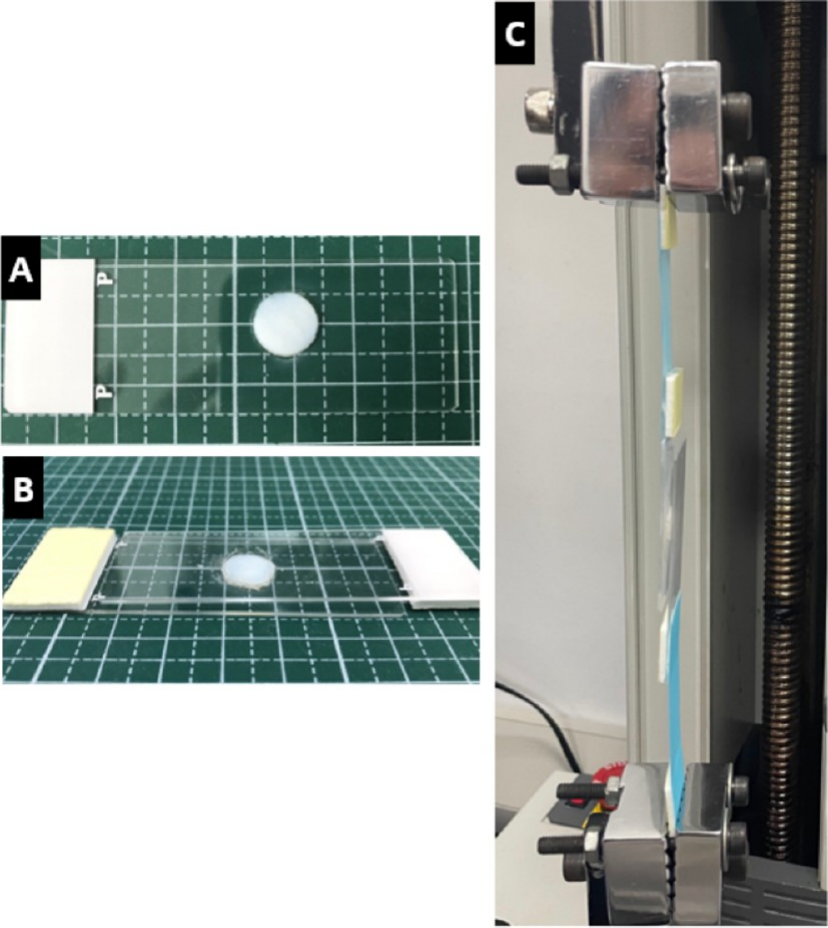
Adhesion test: (A) cartilage disc, (B) cartilage disks stacked with AHAMA/DCP, (C) adhesive testing of AHAMA/DCP using the MTS machine.

Cartilage disks with a 5 mm diameter were fixed onto glass sides. The adhesive strength was measured using a universal testing machine by adding 40 μL of pregel solution to cartilage disks (20 μL each) attached to glass slides and combined with 0, 2, 4, 6, and 8 mg of DCP to produce 0, 5, 10, 15, and 20% (w/v) mixtures. The disks were placed atop one another to sandwich the hydrogel and cartilage powder mixtures. The samples were then exposed to 405 nm ultraviolet (UV) light for 5 min to allow the pregel solution to gel in situ. Then, the samples were pulled to failure (Figure 2).

The universal testing machine was equipped with a load cell of a maximum 50 N capacity with a crosshead speed of 5 mm/min. The adhesive strength was calculated by the failing load (N) at maximum stress (MPa) divided by the bonded area (mm^2^).

#### 2.5.3 Compression Test

A 150 μL of hydrogel was added into a mold and added with 7.5, 15, 22.5, and 30 mg of cartilage powder to form 5, 10, 15, and 20% (w/v) mixtures. The samples were then exposed to 405 nm UV light for 1, 3, 5, and 10 min. After the samples were prepared, the dimensions were measured and then placed between the compression plates with a load cell of 50 N and compressed to failure.

### 2.6 Cytocompatibility and Migration

#### 2.6.1 Cytotoxicity and Cell Proliferation

Cell counting kit-8 (CCK-8) assay was performed to calculate cell proliferation and viability in DCP and AHAMA hydrogel. The cartilage powders were sterilized by exposure to UV light for 12 h and then soaked in LG-DMEM for 3 days at 37 °C to form 10 mg/mL (P1) and 100 mg/mL (P10) conditioned media, respectively. Separately, 5% DCP/AHAMA molds were soaked in medium (PG). 5000 cells were seeded in 96-well plates with normal medium and allowed to attach for 24 h. Following cell attachment, the normal medium was replaced with the conditioned media to determine cell proliferation. The seeded well plates were washed and the media were changed every 2 days. Prepared CCK-8 assay (1:10 ratio in normal cell medium) was used for colorimetric determination of the cell count at days 1, 3, and 5 of the culture periods. The absorbance at 450 nm was measured using an EMax Plus Microplate Reader (Molecular Devices, CA). The equation below was used to calculate the cell growth rate
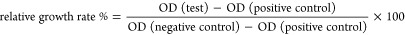
3

#### 2.6.2 Cell Migration

Cell migration in two dimensions was probed by performing a wound healing assay. In this assay, a silicon insert was used to physically separate cells by adhering to the bottom of the dish and preventing cells from growing in the cell-free linear gap. 100 μL of 2 × 10^5^ cells/well were seeded into the silicon inserts on a 24-well plate and incubated at 37 °C overnight. The silicon inserts were removed, and 600 μL of LG-DMEM medium, P1, P10, and PG conditioned media were used to fill the well plate. The cells were monitored and photographed at 0, 6, 12, and 24 h as they moved into the gap separating the two wells using a light microscope equipped with a camera for imaging.

### 2.7 3D Cell Culture and Chondrogenic Differentiation Assessment

The IFPSCs were resuspended in AHAMA and photoinitiator to obtain cell suspension at a density of 3 × 10^5^ cells/mL. Then varying weights of DCP scaffolds were added to 180 μL of AHAMA to form 0, 10, 15, and 20% (w/v) DCP/AHAMA in 7 mm diameter × 4 mm thickness mold. The cells in the hydrogel disks were cultured in two types of media (control and chondrogenic) at 37 °C and 5% CO_2_ for 21 days and the media were changed every 2 days. LG-DMEM was used as the control medium and the chondrogenic medium was prepared by supplementing α-MEM (A1049001, Thermo Fisher Scientific Inc.) with 10^–7^ M dexamethasone, 50 μg/mL ascorbic acid, 1 mM sodium pyruvate (Invitrogen), and 10 ng/mL transforming growth factor-β 3 (TGF-β3, R&D Systems).

After culturing for 21 days, the viability of cells in hydrogels was analyzed using the live/dead staining assay. The samples were dyed with live/dead solutions containing 2 μM calcein-AM (λex/λem: 498 nm/517 nm) and 4 μM EthD-1 (λex/λem: 590 nm/618 nm) for 40 min. The stained cells were visualized using a fluorescence microscope (IX71, Olympus, Japan) with the appropriate filters. The cross sections were deparaffinized and stained with hematoxylin and eosin dyes (HE staining) for cell morphology and safranin-O dye for glycosaminoglycan. In addition, the sGAG content in each digestion solution was measured using DMMB dye assay. The expression of genes encoding collagen I, collagen II, SOX-9, and aggrecan was analyzed by a real-time polymerase chain reaction (real-time PCR).^40^ After the RNAs were extracted using the Trizol reagent, the reaction was performed using the GoTaq 1-Step reverse transcription quantitative real-time polymerase chain reaction (RT-qPCR) kit and StepOnePlus Real-Time PCR System (Applied Biosystems) for 50 cycles. The gene expression levels relative to GAPDH, a housekeeping gene used as an endogenous control, were calculated by using the comparative Ct method, and two-dimensional (2D) cultured IFPSCs were set as the reference. The primer sequences were selected based on previous studies (Table 1).^41−43^ Three samples in each group were measured to calculate the means and standard deviations (SD).

**Table 1:** Sequence of Primers Used for Quantification of Functional Gene Expression

gene	forward	reverse
GAPDH	GGTGAAGGTCGGAGTGAACG	AGTTAAAAGCAGCCCTGGTGA
COL2A	ACACTGCCAACGTCCAGATG	GTGATGTTCTGGGAGCCCTC
ACN	TCTACCGCTGTGAGGTGATGC	TTCACCACGACCTCCAAGG
COL1A	ATGGATGAGGAAACTGGCAACT	GCCATCGACAAGAACAGTGTAAGT
SOX9	GTACCCGCACCTGCACAAC	TCCGCCTCCTCCACGAAG

### 2.8 Statistical Analysis

All data were obtained from independent tests and presented as mean ± standard deviation (SD). Comparison between the values obtained from the study were analyzed using one-way analysis of variance (ANOVA), and Turkey’s post hoc test was used across comparison groups. The significance level was set at 95% (*P* < 0.05), and GraphPad Prism 5.0 software package (GraphPad Software Inc., CA) was used for data analysis.

## 3 Results

### 3.1 Cartilage Decellularization

**Figure 3:**
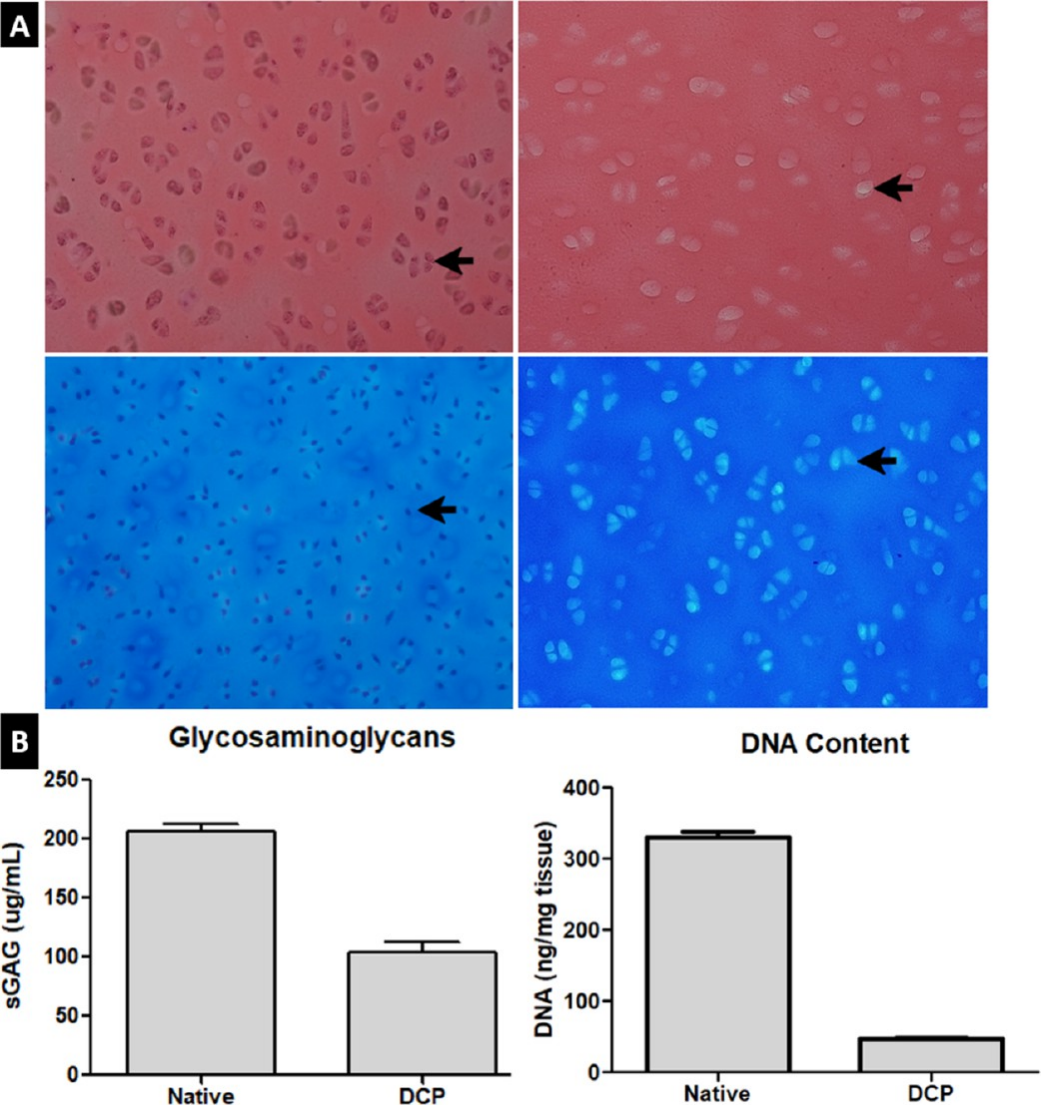
Histological and biochemical assessment of porcine cartilage tissue before and after decellularization. (A) H&E and Masson’s trichrome stain. (B) Glycosaminoglycan and DNA quantification. Scale bars: 100 μm.

H&E staining of the native and decellularized cartilage (Figure 3) shows that the combination of physical, chemical, and enzymatic treatments used resulted in almost complete removal of cells in porcine cartilage. The decellularization process removed 85.6% of the DNA and retained 47.5 ng/mg of extracellular matrix (ECM) dry weight. On the other hand, sGAG was measured using the DMMB dye assay and measured using an ELISA reader. sGAG was reduced by 49.6% after decellularization.

### 3.2 Fabrication of AHAMA Hydrogel

#### 3.2.1 Swelling Ratio

**Figure 4:**
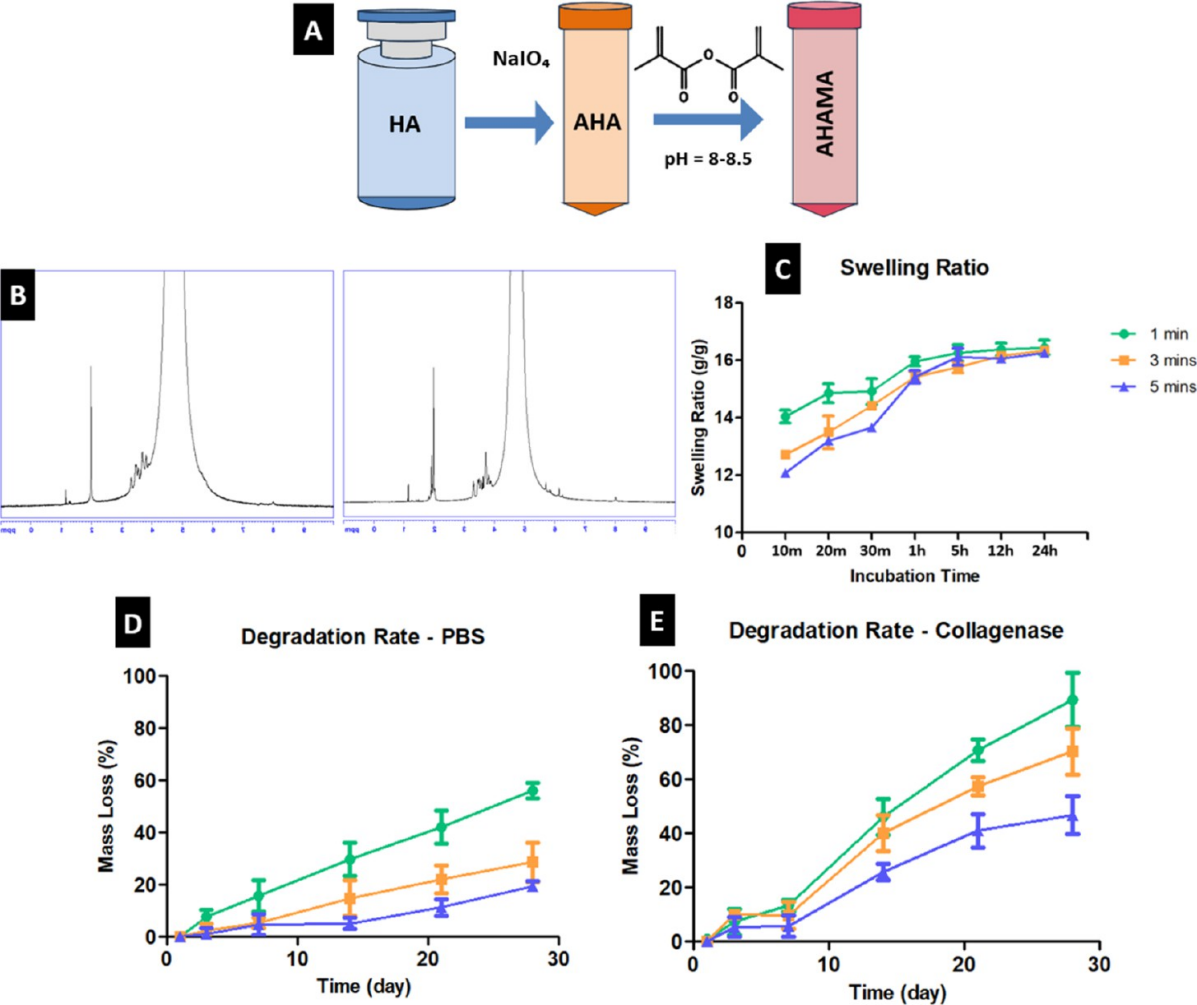
Characterization of fabricated AHAMA hydrogel. (A) Overall reaction for AHAMA synthesis; (B) ^1^H NMR spectra of HA and AHAMA hydrogels in D_2_O (500 MHz); (C) swelling ratio; and degradation properties in (D) PBS and (E) collagenase.

The swelling ratio of AHAMA hydrogels prepared by 1, 3, and 5 min of UV exposure is shown in Figure 4C. The mean swelling ratio of AHAMA at the first 10 min of observation is 12.08 and continues to increase up to 5 h of immersion. The equilibrium swelling of the hydrogel was reached at 5 h of immersion in PBS and remained the same up to 24 h of the observation period. After 24 h, AHAMA had a mean swelling ratio of 16.27, which indicates water uptake 16 times its dry weight.

#### 3.2.2 Enzymatic Degradation

The biodegradability of the hydrogels was examined in the presence of collagenase and compared with their degradation in normal PBS. The weight loss rate after treatment with collagenase is shown in Figure 4D,4E. After 4 weeks, the mass losses in PBS for 1, 3, and 5 min are 56.19, 28.67, and 19.32%, while the mass losses in collagenase are 89.33, 70.33, and 46.7%, respectively. Although there is no significant difference between the groups, the degradation rates in both PBS and collagenase show that the hydrogels with more UV exposure time were degraded more slowly.

#### 3.2.3 Protein Adsorption

The protein adhesion onto the AHAMA hydrogels was carried out by a Micro BCA assay kit, and serial concentrations of commercial bovine serum albumin were used as control. The protein concentrations of the 1, 3, and 5 min groups were 147.88 ± 5.92, 142.96 ± 6.86, and 145.10 ± 7.42 μg/mL, respectively, showing no significant difference between each group. The result indicated that the UV exposure time did not influence the protein adhesive ability.

### 3.3 Mechanical Analysis

#### 3.3.1 Morphological Evaluation and SEM

**Figure 5:**
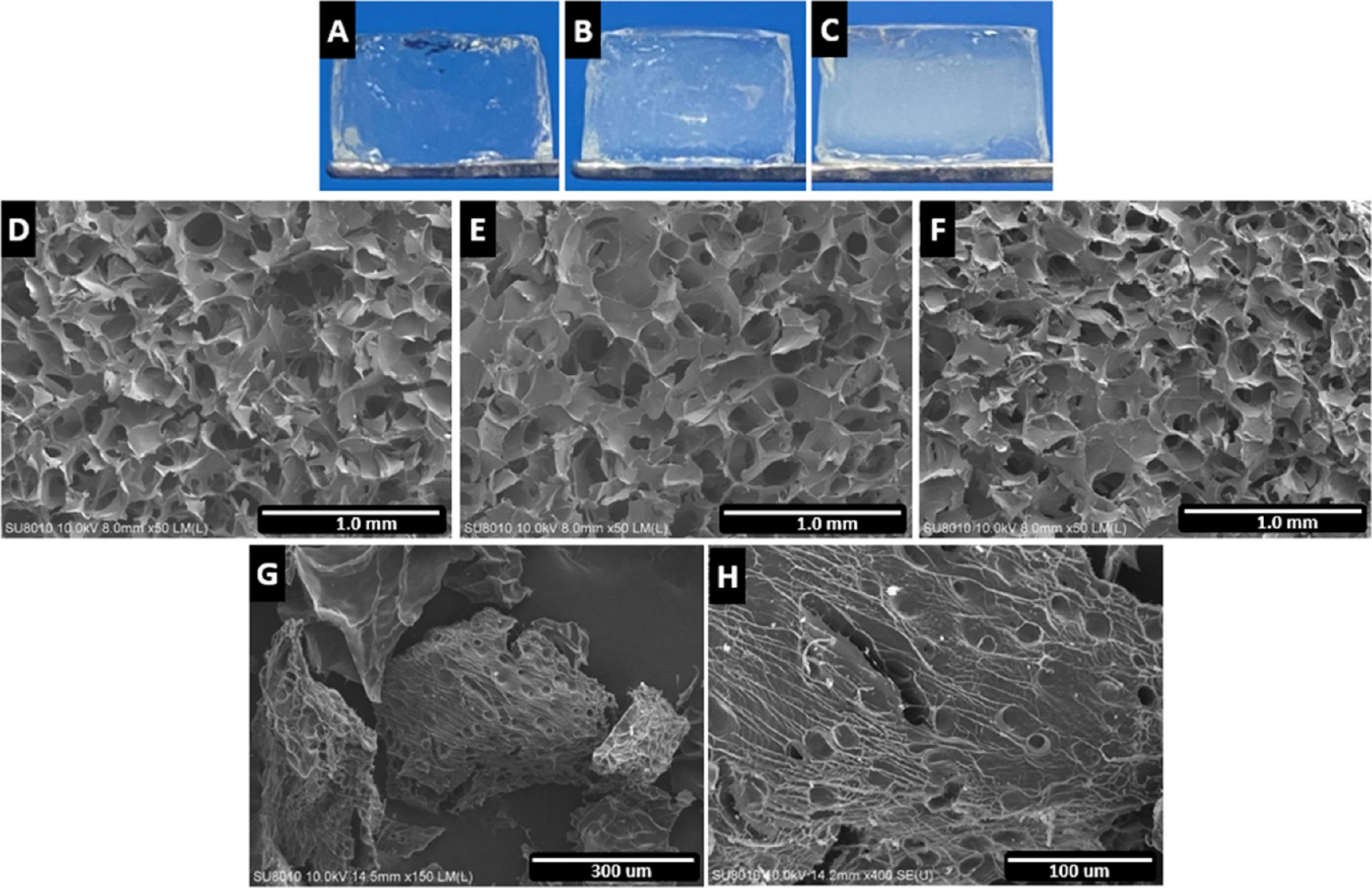
(A–C) Macroscopic appearance and (D–F) SEM images of the inner structure of AHAMA prepared by 1, 3, and 5 min of UV exposure. Scale bar = 1 mm. SEM images of decellularized cartilage powder at (G) 150× and (H) 400× magnification. Scale bars = 300 and 100 μm.

After 1, 3, and 5 min of UV exposure, macroscopic appearances were observed in three different experimental groups (Figure 5A–C), and images were captured using a digital camera. All groups gelled successfully and kept cylindrical shapes with 7 mm diameter and 4 mm height. The hydrogel formed by 1 min UV exposure appeared transparent, soft, and weak; while increasing the exposure time to 3 and 5 min, the hydrogels appeared more translucent, tougher, and more rigid. The microstructure of the three groups was captured by SEM, showing regular distribution of deep and dense pores for nutrient transportation and cell migration. The appropriate pore form and distribution indicate a favorable structure for cell growth within the hydrogel structure (Figure 5D–F).

The SEM micrographs of the decellularized cartilage powder show sizes ranging from 250 to 300 μm. They also demonstrated surface features such as the sites of cell removal and exposure of the fibrillar collagen matrix in the tissue (Figure 5G,H).

#### 3.3.2 Adhesion Test

Porcine cartilage was used to measure the adhesive strength of various concentrations of AHAMA hydrogel and DCP to the tissue. The adhesive strength was calculated by obtaining the failing load at the maximum stress divided by the bonded area. The AHAMA hydrogel alone showed a good mean adhesive strength of 14.86 kPa. Incorporating increasing amounts of DCP increases the adhesive strength to cartilage tissue. The mean values are as follows: 24.24, 44.86, 45.38, and 48.66 kPa for 5, 10, 15, and 20% (w/v) DCP/AHAMA, respectively.

**Figure 6:**
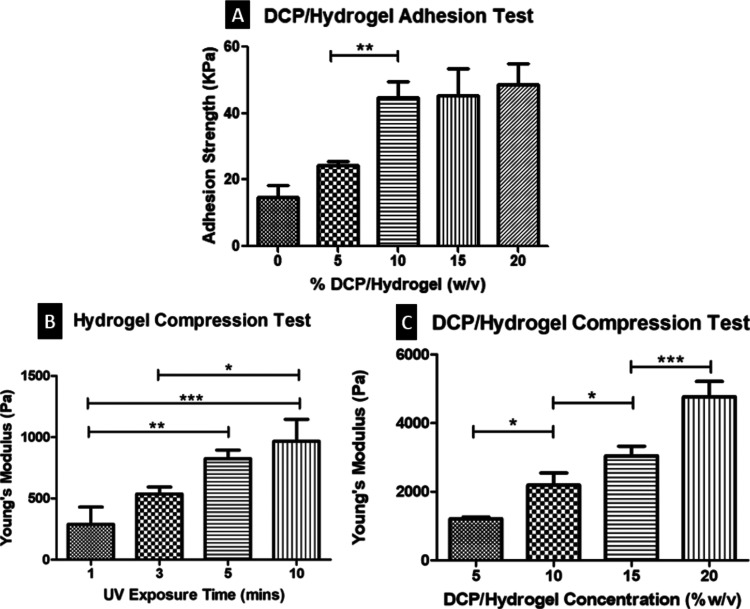
Mechanical properties of DCP/AHAMA hydrogels. (A) Adhesion strength; (B) Young’s modulus for 1, 3, 5, and 10 min UV exposure; (C) Young’s modulus for 5, 10, 15, and 20% (w/v) concentrations. *P < 0.05, **P < 0.01, ***P < 0.001.

The increasing trend shows a significant adhesive strength difference between 10, 15, and 20% (w/v) DCP/AHAMA concentrations compared to hydrogel alone and 5% (w/v) DCP/AHAMA (Figure 6A).

#### 3.3.3 Compression Test

For the compression test, different hydrogel UV exposure times and varying concentrations of DCP/AHAMA disks were compressed to failure. The stress (MPa) vs % strain was plotted to obtain the Young’s modulus. For the hydrogel UV exposure time of 1, 3, 5, and 10 min, the mean Young’s moduli are 288.51, 535.06, 823.81, and 966.72 Pa, respectively (Figure 6B). Increasing the exposure time also increases the Young’s modulus of the hydrogel. On the other hand, different concentrations of DCP/AHAMA molds were prepared. The mean Young’s moduli are 1210.33, 2191.13, 3046.3, and 4765.17 Pa for 5, 10, 15, and 20% (w/v) DCP/AHAMA (Figure 6C).

Different exposure times to UV light increase the compressive strength of the AHAMA hydrogel. Significantly different values can be observed after 5 and 10 min compared to only 1 min of exposure. Similarly, increasing the DCP/AHAMA hydrogel concentration also increases the Young’s modulus. 10, 15, and 20% (w/v) obtained significantly higher values than 5% (w/v) DCP/AHAMA. Notably, 20% (w/v) concentration exhibited a significant compressive strength increase compared to 15% (w/v) DCP/AHAMA hydrogel (Figure 6C), indicating that the addition of more cartilage powder in the mixture improves the compressive strength of the scaffold material.

### 3.4 Cytocompatibility and Migration

**Figure 7:**
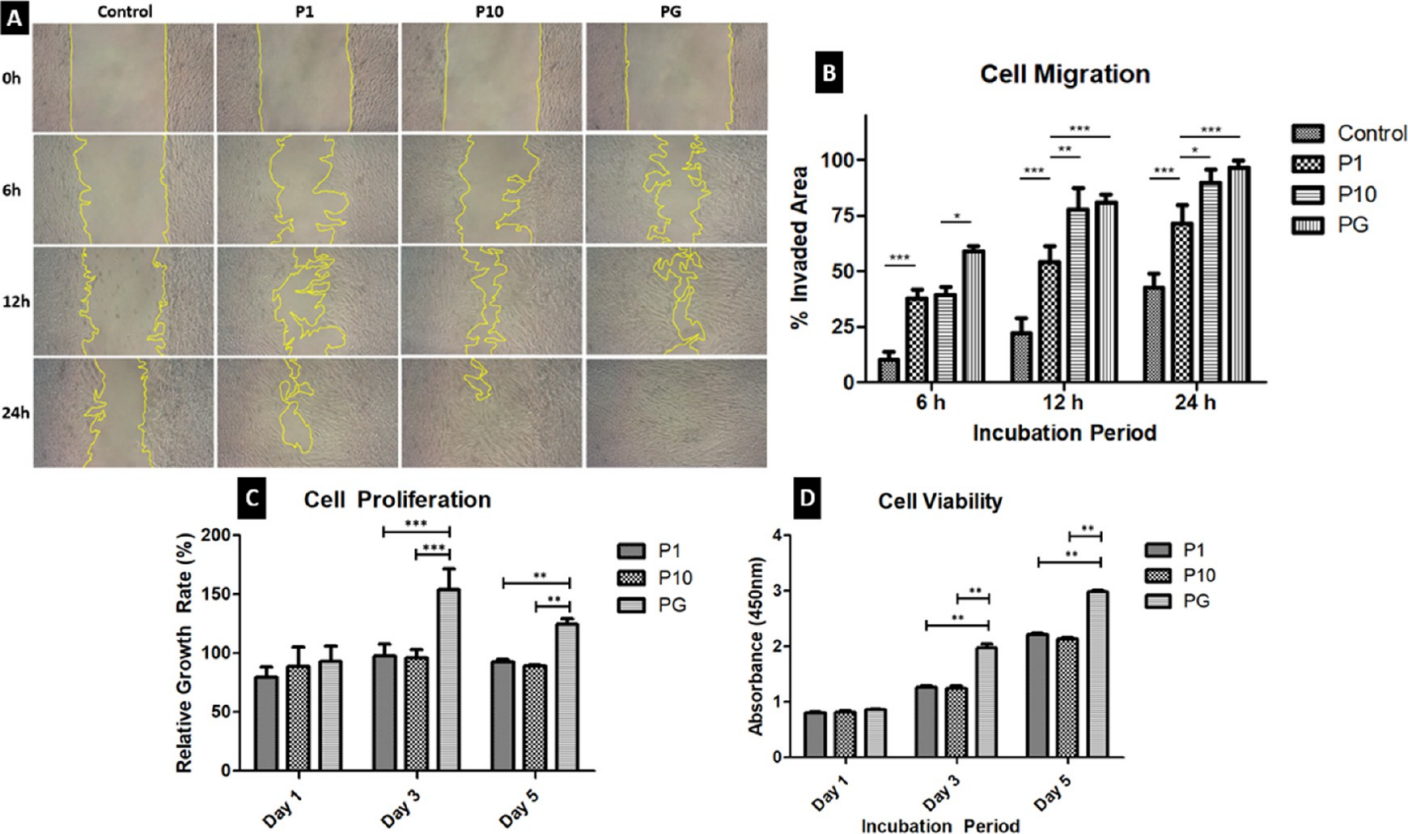
Biocompatibility of DCP and AHAMA hydrogels in vitro. (A, B) Cell migration, (C) cell proliferation, and (D) cell viability assays. *P < 0.05, **P < 0.01, ***P < 0.001.

IFPSCs showed cell movement, causing the gap to close after 24 h for P1, P10, and PG conditioned media (Figure 7A). Compared to the groups supplemented with conditioned cartilage media, the control group exhibited very minimal movement. It did not close the gap in artificially created wounds even after 24 h of incubation. The wounds in the groups treated with 1× and 10× concentrations of conditioned cartilage powder media (P1 and P10) completely closed after 24 h of incubation, but AHAMA hydrogel and DCP conditioned cartilage medium (PG) increased the proliferation and migration of cells and almost healed at 12 h of incubation. Within the 24 h incubation period, the cells reached full confluence in the wound area. At 12 h of incubation, the invaded area by the cells increased significantly for both 1× and 10× groups as compared to the control group, which indicated that the presence of the cartilage powder and hydrogel in the media improved both proliferation and migration (Figure 7B,C).

The cytotoxic effect of DCP and AHAMA hydrogel was evaluated using the CCK-8 assay (Figure 7D). Cells incubated with both concentrations of DCP media have increased cell viability compared to day 1 of analysis. On the third day of incubation, the DCP/AHAMA mixture (PG)-incubated cells had a significant increase in viability compared to both 1× and 10× concentration groups.

### 3.5 3D Cell Culture

#### 3.5.1 Live/Dead Staining

**Figure 8:**
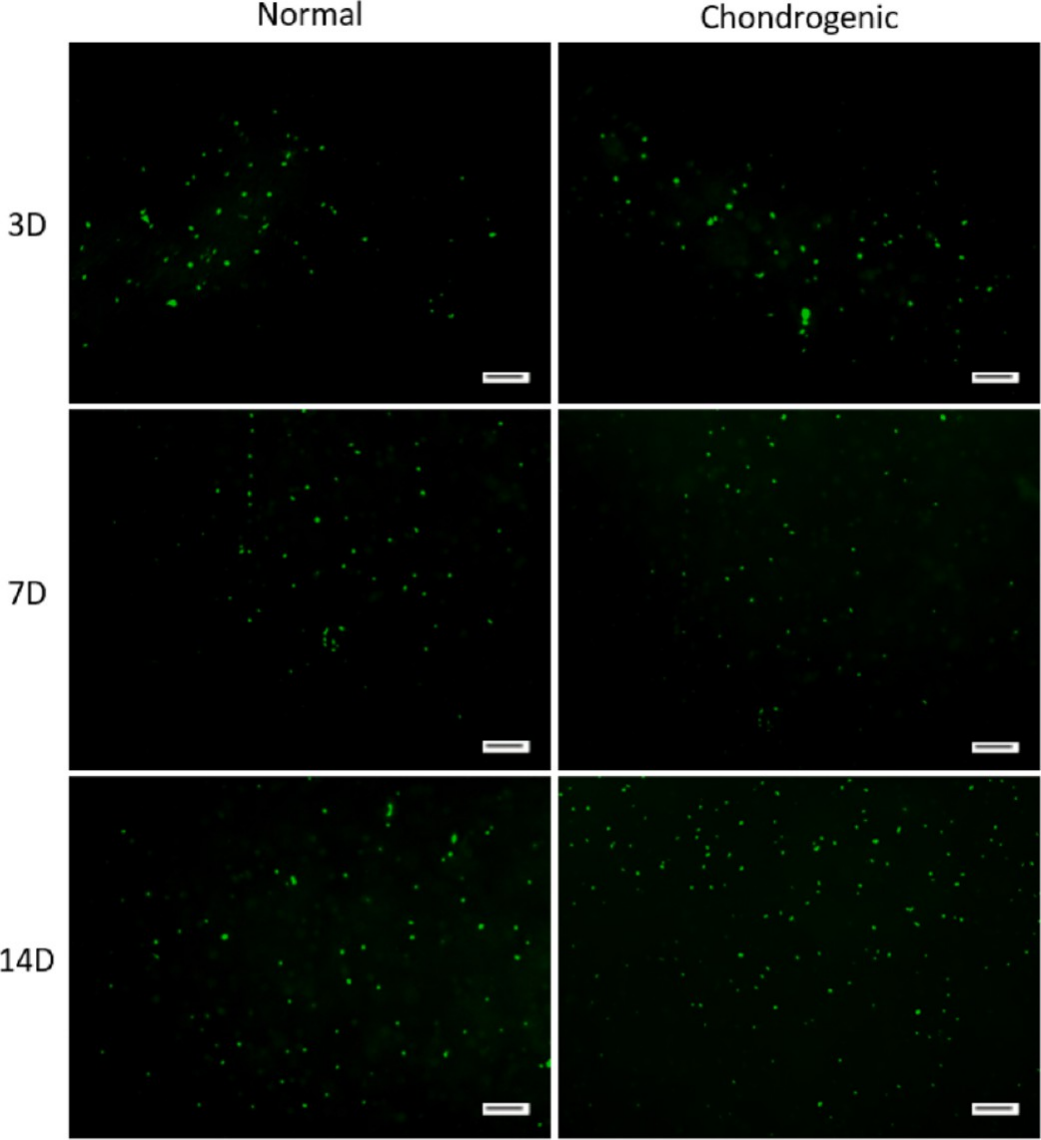
Live–dead staining fluorescent images of MSCs encapsulated in AHAMA after culturing for 3, 7, and 14 days in normal and chondrogenic medium. Calcein-AM for live cells (green) and EthD-1 for dead cells (red). Scale bar: 100 μm.

Cell viability was further investigated by live/dead staining after the IFPSCs were encapsulated in AHAMA hydrogels cultured in normal and chondrogenic media for 3, 7, and 14 days (Figure 8). All groups showed green, indicating live cells and very few dead cells (red). The results indicated that the cells have high cell viability even after photo-cross-linking, showing successful encapsulation in AHAMA hydrogel. Though more cell density can be observed in the chondrogenic group, the cell density of the normal group is comparable, indicating that the AHAMA hydrogel possesses excellent biocompatibility. On the other hand, groups with both DCP and AHAMA cannot be visualized with live/dead stain as the presence of DCP makes the hydrogel appear translucent to opaque, interfering with the samples’ ability to emit fluorescent light.

#### 3.5.2 Histological Staining

**Figure 9:**
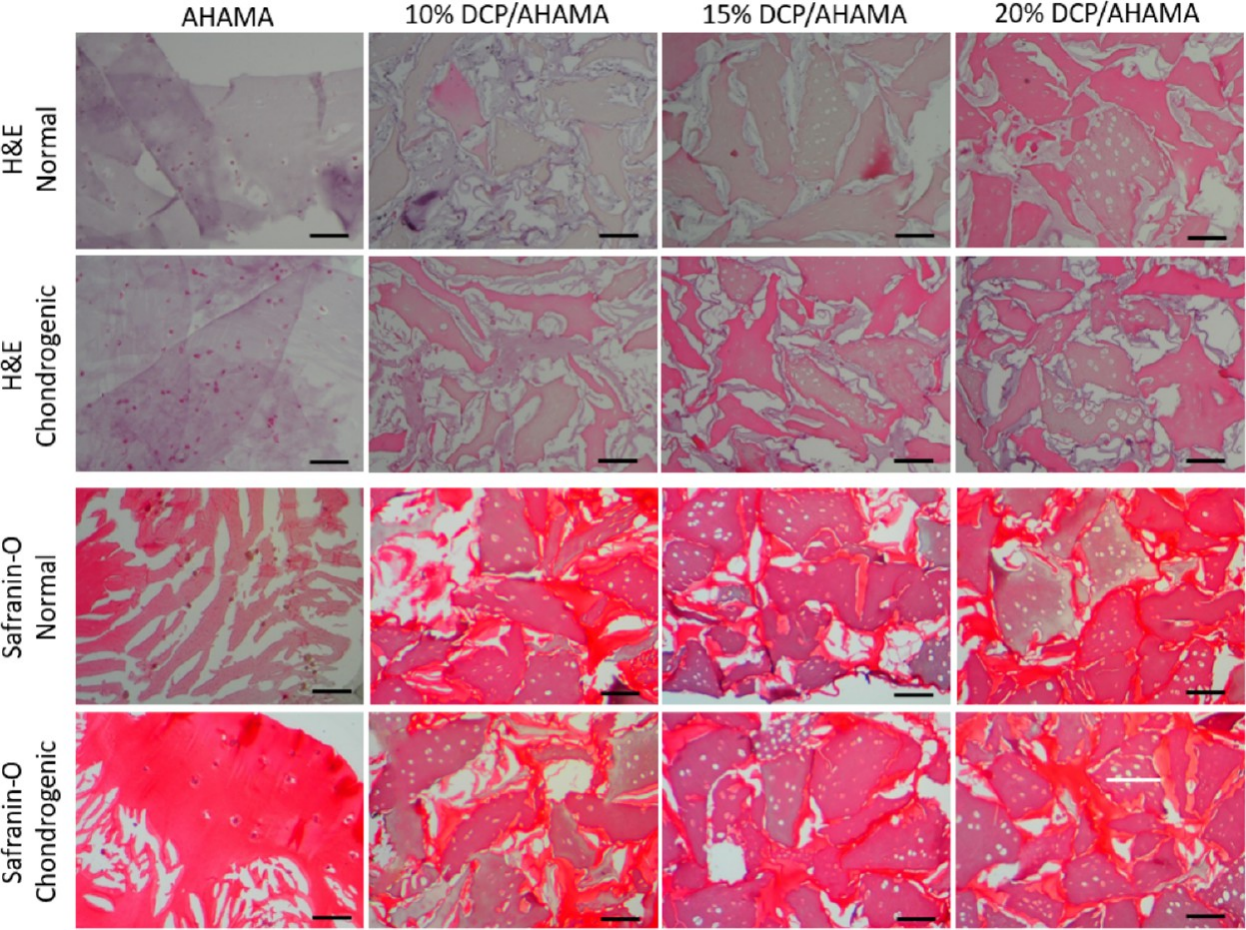
H&E and safranin-O staining of MSCs cultured in DCP/AHAMA hydrogels in normal and chondrogenic media for 21 days.

Cell morphology and cartilaginous matrice production of the IFPSCs after 21 days of culture in the DCP/AHAMA hydrogels were investigated by histological staining (Figure 9). H&E staining indicated a homogeneous cell distribution for all the DCP/AHAMA hydrogels. The cells showed round morphology in 10, 15 and 20% DCP/AHAMA in chondrogenic medium (C2, C3, and C4) and 15 and 20% DCP/AHAMA in normal medium (N3 and N4). The round morphology in embedded cells is an indication of chondrogenic phenotype. Safranin-O staining showed that the cells cultured in DCP/AHAMA in normal media (N2–N4) also showed positive staining of proteoglycans compared to those cultured in chondrogenic media (C2–C4). Meanwhile, the cells cultured in both normal and chondrogenic groups containing AHAMA hydrogels only (N1 and C1) showed less proteoglycans secretion capacity.

**Figure 10:**
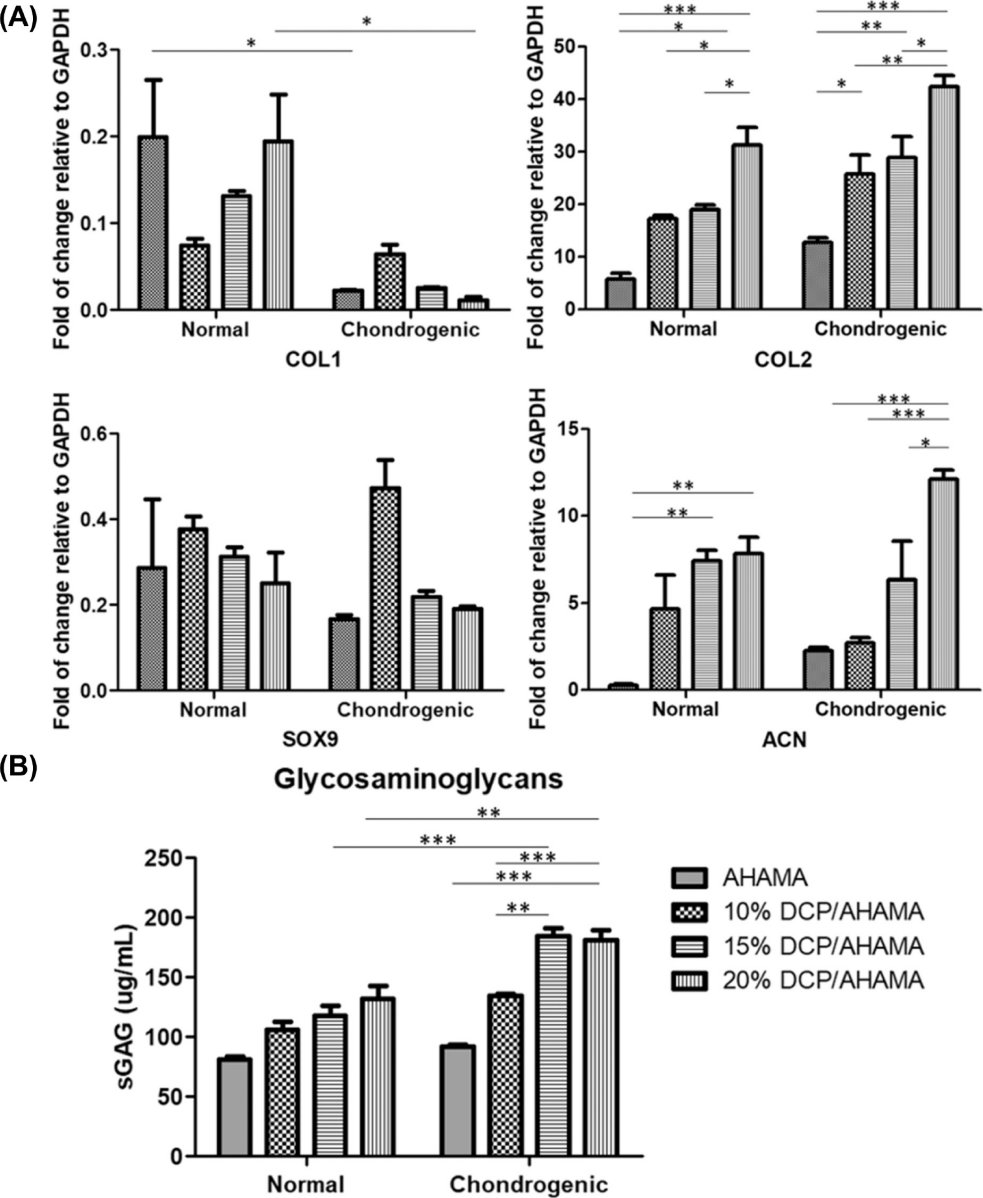
MSC chondrogenesis in DCP/AHAMA hydrogel cultured in normal and chondrogenic media. (A) Expression of genes encoding COL1, COL2, SOX9, and ACN. (B) Quantitative analyses of glycosaminoglycans contents. *P < 0.05, **P < 0.01, ***P < 0.001.

The secretion of ECM proteins is essential for cartilage regeneration because abundant ECMs such as sGAG surround chondrocytes in cartilage. The sGAG (μg/mL) was measured using the DMMB dye assay. The sGAG was significantly higher in the presence of DCP/AHAMA in both groups than in the AHAMA hydrogel alone. The graph shows that increasing the DCP content also increases the sGAG calculated. On the other hand, significant differences in the values in N3 vs C3 and N4 vs C4 were observed (Figure 10B).

#### 3.5.3 Gene Expression

The expression of genes encoding COL1, COL2, SOX9, and ACN was analyzed by real-time PCR (Figure 10A). Cells cultured in chondrogenic medium expressed less COL1 and there was no significant difference in SOX9 expression compared to the cells grown in normal medium. For the genes encoding cartilaginous matrices, COL2 and ACN, significant differences can be observed as the concentration of the DCP/AHAMA hydrogel increases. As expected, the measured values in the chondrogenic group were higher than those in the normal group. But comparing the normal group with 20% DCP/AHAMA (N4) to the chondrogenic groups with AHAMA, 10% DCP/AHAMA, and 15% DCP/AHAMA (C1, C2, and C3), there is higher expression of COL2 and ACN, indicating that the addition of DCP to AHAMA in higher concentration has good expression of the chondrogenic phenotype, even higher than that of lower concentration hydrogel groups cultured in chondrogenic medium.

## 4 Discussion

The use of decellularized cartilage is advantageous because it retains the biocompatibility, as well as structural and functional proteins while inhibiting immune response due to the removal of cellular components.^44^ In this research, porcine cartilage was decellularized by combining physical, chemical, and enzymatic methods. Because the cartilage has a dense structure, physical breakdown of the tissue, such as breaking down into fragments or particles, is performed to increase the surface area and permeability of chemical agents.^18,45^ However, subjecting through these physical processes can destroy the unique heterogeneous structure of the cartilage tissue.^46^ The decellularization efficiency was assessed by histological analysis and assessment of the DNA content (Figure 3). H&E and Masson’s trichrome staining of native and decellularized articular cartilage groups revealed that while retaining almost the same collagen content, the treatment procedure reduced DNA by 85.6%, retaining only 47.5 ng/mg ECM dry weight and no visible nuclear material in the H&E stain (Figure 3A). This shows that the decellularization of the porcine cartilages was successful as the results meet the criteria for sufficient decellularization.^47^ Another chondroprotective component of the cartilage, glycosaminoglycans (GAG), was measured using the DMMB dye assay. In a previous study using decellularization by Triton X-100 and incubating the tissues in a solution of DNase and RNase,^48^ the measured sGAG was significantly reduced to about 20%. In this study, the retained sGAG was kept at 50% of the native tissue (Figure 3B), proving to have used a more efficient combination of decellularization methods.

Another scaffold used in this research is modified HA methacrylate hydrogel with aldehyde groups (AHAMA), which was fabricated according to the previous research performed by Chen et al.^38^ HA is widely used in cartilage regeneration since it is known to have good biocompatibility, promote the proliferation of chondrocytes^27^ and MSCs,^28^ and promote cartilage regeneration.^29^ HA can be modified by aldehyde groups to facilitate adhesion, and the incorporation of methacrylate enables the AHAMA to self-gel.^38^ The ^1^H NMR spectra of the fabricated AHAMA showed two new peaks, at around 6 ppm corresponding to the C=C bond from methacrylate formation and around 3.6 ppm from the aldehyde formation (Figure 4B). This study adopted the preparation and synthesis method performed by Chen et al.,^38^ where the methacrylate modification level of AHAMA was estimated to be 24% and the oxidation level was 36%. The AHAMA hydrogel could take up to 16 times its dry weight, indicating the scaffold’s excellent water absorption without structural destruction (Figure 4C). Materials with high water uptake and swelling ratios act as physical cues to promote chondrogenic differentiation.^49^ A previous study showed that an increase in the swelling ratio positively affects the chondrogenic differentiation of mesenchymal stem cells.^50^

Degradability is a vital feature of hydrogels. The degradation of HA-based hydrogels affects the bone healing rate and organization of new collagen.^51^ The required hydrogel degradation rate differs based on its application. For articular cartilage, a hydrogel that degrades slowly is more suitable.^52^ The degradation of the groups showed a stable rate in the first week. An abrupt increase in mass loss can be observed in the 1 min group for both PBS and collagenase solutions on the second week, losing about 30 and 45%, respectively. On the third and fourth weeks, the mass losses in collagenase for groups 1, 3, and 5 min are 70.71 and 89.33%, 57.52 and 70.33%, and 40.9 and 46.7%, respectively. The mass losses of the 1 min group in both PBS and collagenase are considerable due to less dense structure as a result of less cross-linking time. The group UV exposed for 5 min maintained a stable degradation rate from 3 to 4 weeks and showed the slowest and most suitable degradation property in the presence of collagenase (Figure 4D,E). These results suggested that the UV exposure time during preparation could control the enzymatic degradability of AHAMA hydrogels.

For protein adhesion, there is no significant difference observed between the groups, and the result indicated that UV exposure time did not influence the protein adhesive ability; the embedded or recruited cells interact with AHAMA gels in the same manner.

The macroscopic appearance of the hydrogels using different UV exposure times (1, 3, and 5 min) resulted in different cross-linking densities. Shorter UV exposure time, 1 and 3 min, resulted in softer, weaker, and more transparent hydrogels. Whereas the 5 min group resulted in more translucent and palpable hydrogels and much easier handling for in vitro tests (Figure 5A–C). HA hydrogels’ cross-linking density affects chondrogenesis, matrix deposition, and hypertrophy in encapsulated MSCs. Also, shorter UV exposure time results in partial consumption of methacrylate, leaving some of it unreacted.^53^ The microstructures observed using SEM showed a regular distribution of deep and dense pores for nutrient transportation and cell migration. The appropriate pore form and distribution indicate a favorable structure for cell growth within the hydrogel structure (Figure 5D–F).

In addition to influencing the shape and metabolic activity of the engineered construct, scaffolds must also be capable of withstanding the mechanical environment of the native tissue that is to be replaced.^54,55^ The compressive strength of the scaffolds was measured using a mechanical testing system prepared with varying exposure time to UV (Figure 6B) and different concentrations of the DCP/AHAMA hydrogel mixture (Figure 6C). The calculated Young’s modulus of the stress–strain curve during 10–20% strain shows an increasing trend for both setups. Both 5 and 10 min exposure times are significantly higher than 1 min exposure, but no significant difference between 5 and 10 min. As for the concentration, both 15 and 20% (w/v) DCP/AHAMA have significantly higher values than the other groups, while 20% (w/v) obtained significantly higher mean Young’s modulus than 15% (w/v).

Porcine cartilage was used to measure the adhesive strength of the AHAMA hydrogel to tissue (Figure 6A). AHAMA hydrogel alone obtained a mean lap shear strength of 14.86 kPa. Upon addition of various weights of DCP to form 5, 10, 15, and 20% (w/v) DCP/AHAMA, the lap shear strength also increased and reached a mean of 48.56 kPa. Though there is no significant difference in values obtained for 10, 15, and 20%, an increase in the concentration of DCP/AHAMA showed higher adhesive strength to porcine cartilage tissue. Adding DCP to the hydrogel improves the reported adhesive strength of AHAMA alone.^38^

The issue of biocompatibility may arise due to aldehyde groups on oxidized hyaluronic acid, but this depends on the material concentration. Literature that used hydrogels with aldehyde groups in cartilage repair reported no cytotoxicity.^56,39,57^ In addition, methacrylated HA hydrogels provided excellent microenvironment for chondrogenesis and growth factor delivery for cartilage tissue engineering applications.^58−60^ In this study, aldehyde groups with methacrylated HA gels had negligible cytotoxic effects. Cell adhesion, proliferation, and viability are inversely related to the cytotoxic effects of DCP and AHAMA on the cells (Figure 7). Samples containing both DCP and AHAMA cannot be visualized using live/dead staining because the presence of DCP causes the hydrogel to appear translucent to opaque. The opacity of materials presents a barrier to light-based observations. For instance, in one study, the polypeptide hydrogel used becomes opaque at body temperature, hindering the assessment of encapsulated cells.^61^ Another study noted limitations in assessing cell morphology and behavior due to light attenuation and restricted penetration caused by media turbidity during illumination and fluorescence studies. Currently, this challenge persists in imaging opaque samples and requires improvements to enhance light transmittance.^62^ In our study, the assessment of cytocompatibility was similarly impacted by the limited light transmittance of the samples during fluorescent microscopy. Instead, robust quantitative results were obtained using CCK-8 assay as shown in Figure 7C,7D, and visual confirmation of viable cells after encapsulation was provided by the live/dead staining images (Figure 8).

The chondrogenic potential of the DCP/AHAMA hydrogel was investigated by cell encapsulation for 3 weeks in vitro. The combination of DCP and AHAMA hydrogel promotes chondrogenic differentiation, mimics the cartilage structure, and promotes the survival of IFPSCs and the production of GAGs. Moreover, the combination of DCP and AHAMA enhanced the chondrogenic differentiation of cells, as evidenced by increased COL2 and ACN expression (Figure 10). The chondrogenic medium was supplemented with TGF-β3, a known agonist of chondrocyte differentiation.^63^ Therefore, the results showed an expected higher expression of chondrogenic markers than that of the IFPSCs cultured in DCP/AHAMA hydrogels in normal medium. In a previous study, heparin and HA, in combination with TGF-β3, enhanced chondrogenesis.^64^ In an in vivo study, HA hydrogel, TGF-β3, and chondrocytes were implanted into mice, which resulted in more production of glycosaminoglycan and collagen compared with mice treated with hydrogels alone.^65^ In the normal groups with high concentrations of DCP/AHAMA, particularly 20%, the expressions of chondrogenic markers are comparable to those treated with chondrogenic medium (C1, C2, and C3). These results signify that adding DCP to AHAMA at a higher concentration enhances the expression of the chondrogenic phenotype even better than that of lower concentration hydrogel groups cultured in chondrogenic medium.

Similarly, lower levels of COL1 were observed in IFPSCs cultured with hydrogels in chondrogenic medium compared to hydrogels in normal medium, but lower COL1 can be observed in high DCP concentration in normal medium compared to AHAMA hydrogel alone, suggesting the role of decellularized extracellular matrix in blocking chondrocyte hypertrophy.^66^

## 5 Conclusions

Decellularized cartilage powder (DCP) was successfully obtained by subjecting porcine cartilage to physical, chemical, and enzymatic procedures. Hyaluronic acid (HA) hydrogel modified by aldehyde groups and methacrylate (AHAMA) was fabricated to provide tissue adhesion. The mechanical properties obtained by combining these two, specifically, 5 min of UV exposure and 20% (w/v) DCP/AHAMA showed high tissue adhesion and good compressive strength. In vitro tests showed that the combination of DCP and AHAMA mimics the cartilage structure, promotes the survival of cells, and enhances the GAG production and chondrogenic differentiation of IFPSCs. Overall, DCP/AHAMA recapitulates the complex biomechanical properties and microenvironment of native cartilage, making this combination suitable for cartilage tissue engineering applications. Through a combination of further in vitro experiments, such as the introduction of signaling factors and initial in vivo studies in small animal models, the biocompatibility and regenerative capacity of the combination of DCP and AHAMA can be affirmed.
